# Research Progress of Polysaccharide-Gold Nanocomplexes in Drug Delivery

**DOI:** 10.3390/pharmaceutics16010088

**Published:** 2024-01-09

**Authors:** Ming Song, Adila Aipire, Elzira Dilxat, Jianmin Li, Guoyu Xia, Ziwen Jiang, Zhongxiong Fan, Jinyao Li

**Affiliations:** 1 Institute of Materia Medica & College of Life Science and Technology, Xinjiang University, Urumqi 830017, China; 107552100985@stu.xju.edu.cn (M.S.); adila16@xju.edu.cn (A.A.); 107552100963@stu.xju.edu.cn (E.D.); 107552201047@stu.xju.edu.cn (J.L.); xgy1823@stu.xju.edu.cn (G.X.); 2 Department of Gynecology, Beijing Obstetrics and Gynecology Hospital, Capital Medical University, Beijing Maternal and Child Health Care Hospital, Beijing 100006, China; jiangziwen@ccmu.edu.cn

**Keywords:** polysaccharides, gold nanoparticles, drug delivery, nanocarriers, oncotherapy

## Abstract

Clinical drug administration aims to deliver drugs efficiently and safely to target tissues, organs, and cells, with the objective of enabling their therapeutic effects. Currently, the main approach to enhance a drug’s effectiveness is ensuring its efficient delivery to the intended site. Due to the fact that there are still various drawbacks of traditional drug delivery methods, such as high toxicity and side effects, insufficient drug specificity, poor targeting, and poor pharmacokinetic performance, nanocarriers have emerged as a promising alternative. Nanocarriers possess significant advantages in drug delivery due to their size tunability and surface modifiability. Moreover, nano-drug delivery systems have demonstrated strong potential in terms of prolonging drug circulation time, improving bioavailability, increasing drug retention at the tumor site, decreasing drug resistance, as well as reducing the undesirable side effects of anticancer drugs. Numerous studies have focused on utilizing polysaccharides as nanodelivery carriers, developing delivery systems based on polysaccharides, or exploiting polysaccharides as tumor-targeting ligands to enhance the precision of nanoparticle delivery. These types of investigations have become commonplace in the academic literature. This review aims to elucidate the preparation methods and principles of polysaccharide gold nanocarriers. It also provides an overview of the factors that affect the loading of polysaccharide gold nanocarriers with different kinds of drugs. Additionally, it outlines the strategies employed by polysaccharide gold nanocarriers to improve the delivery efficiency of various drugs. The objective is to provide a reference for further development of research on polysaccharide gold nanodelivery systems.

## 1. Introduction

**Scheme 1:**
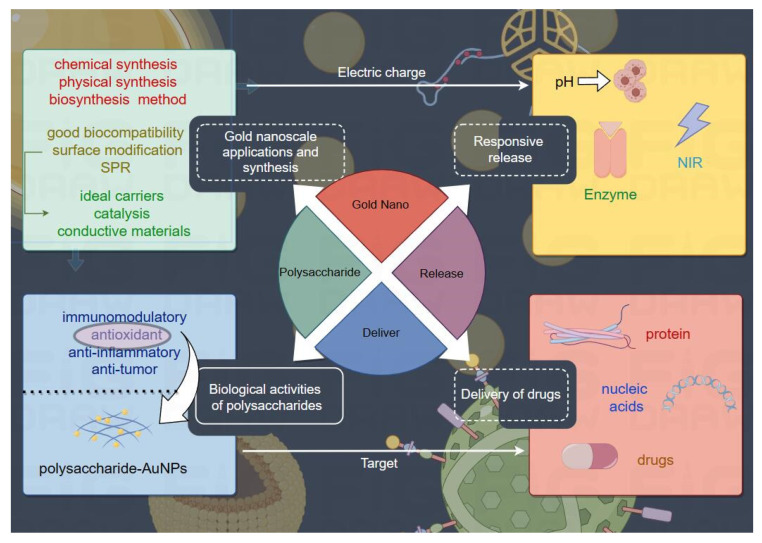
Application summary of polysaccharide and gold nanoparticles in drug delivery and release. The associated image materials were prepared by Figdraw 2.0.

Water solubility and stability are indeed significant factors that impede the clinical application of various drugs. In order to overcome these challenges, carrier-based drug delivery systems (DDSs) have been developed. Such systems have shown promise as an effective strategy to enhance therapeutic efficiency due to their ability to achieve high drug-loading capacities and facilitate sustained drug release [1]. Drug delivery systems refer to the use of carriers to transport drugs and control their release and distribution within the human body. These systems are designed to enhance the specificity and utilization of drugs while minimizing harm to normal tissues. By loading drugs onto carriers, DDSs can effectively deliver drugs to specific locations in the body and regulate their release rate, optimizing the therapeutic effect and reducing potential side effects. DDSs employs a variety of carriers, such as nanoparticles, liposomes, and micelles, to encapsulate drugs and protect them from degradation. These carriers can be designed to target specific cells or tissues through surface modifications or active targeting strategies, allowing for precise drug delivery. Once the carriers reach the desired location, the drug can be released in a controlled manner, either through diffusion, degradation, or external triggers. This controlled release ensures that the drug reaches its intended target and maintains a therapeutic concentration for a prolonged period. Moreover, DDSs allow for the customization of drug release profiles, enabling tailored therapy for individual patients. For instance, some drugs may require sustained release over an extended period, while others may require a burst release for immediate action. DDSs can accommodate these diverse requirements through the design of carrier properties, such as particle size, surface charge, and composition. In the first 40 years of drug delivery technology’s existence (1950–1990), significant advancements were made. However, it was not until the introduction of the first polyethylene glycolized protein in 1990 that the era of polyethylene glycol began. This breakthrough had a profound impact on the development of liposomes and lipid nanoparticle formulations, and it drew considerable attention to nanodelivery systems [2]. This review primarily focuses on the research progress of polysaccharide gold nanoparticles as drug delivery systems and discusses their potential future applications (Scheme 1). The rest of the manuscript will delve into the synthesis methods, characterization techniques, and various applications of these nanoparticles in drug delivery. Additionally, the review will discuss the challenges and opportunities associated with using polysaccharide gold nanoparticles in the medical field. By examining the current research and discussing potential future developments, this review aims to provide a comprehensive understanding of the role of polysaccharide gold nanoparticles in drug delivery systems.

**Figure 1:**
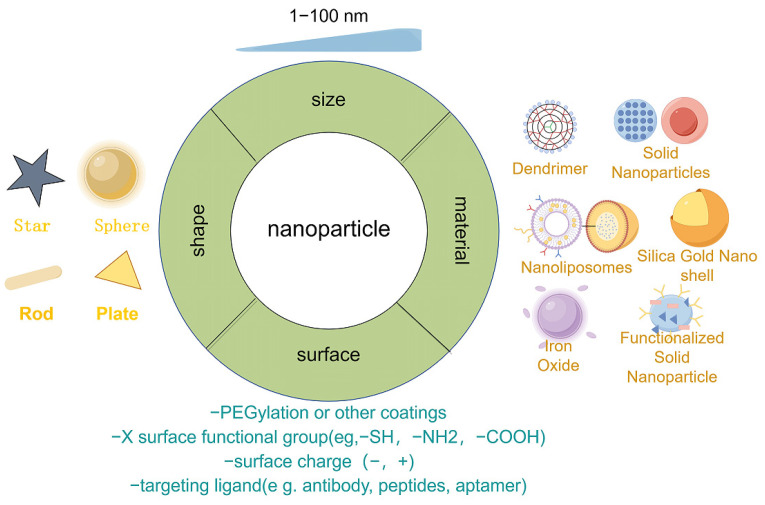
Application summary of nanodelivery systems. The associated image materials were prepared by Figdraw 2.0.

Nanocarriers are a novel drug delivery system with the ability to efficiently transport drugs to the intended target site. They possess a larger specific surface area, allowing for a higher drug load, thereby increasing the concentration of released drugs at the designated site. This enhanced drug release rate makes nanocarriers the optimal choice for drug delivery in current times (Figure 1). Nano-drug delivery systems (Nano-DDSs) have shown promise in improving drug effectiveness by targeting diseased cells. Multifunctional DDSs are tailored to deliver drug molecules selectively to the desired tissue or organ using passive and active targeting mechanisms, enhancing accumulation, retention, and overall efficacy [3]. Nano-drug delivery systems include targeted nanocarriers and stimulus-responsive nanocarriers. Targeted nanocarriers include passive and active nanoparticle delivery systems. Active targeting promotes active cellular uptake of nanoparticles by cancer cells, whereas passive targeting allows nanoparticles to be efficiently localized in a tumor microenvironment [4]. Passive targeting refers to the accumulation of small molecules at the tumor site through the EPR effect. Due to the EPR effect, nanocarriers with a particle size of less than 200 nm can easily penetrate the tumor vasculature system, resulting in passive targeting, which produces therapeutic effects in the tumor tissue. Passively targeted drug delivery systems mainly include liposomes, microemulsions, and nanoparticles [5]. In addition, combining nanocarriers with pH-dependent polymers can be utilized to target tumor cells actively, facilitating intracellular drug release from cancer cells [6]. Active targeting means modifying the surface of nanoparticles using the targeting ligands to direct to the specific receptors overexpressed on tumor cells precisely. The targeted ligands include antibodies, polysaccharides, and proteins, among others. The active targeting nanoparticles passively exude blood vessels into tumor tissue [7]. This recognition stimulates receptor-mediated endocytosis, which in turn internalizes nanocarriers and enhances their ability to exert therapeutic effects on tumors [8]. Several classifications of nanomedicine delivery systems can be grouped into three types based on the drugs they deliver: nanosystems for transporting chemical drugs, nanosystems for transporting peptide- or protein-based agents, and nanosystems for transporting nucleic acids.

Furthermore, these nanodelivery systems can be categorized based on the nanocarrier used, including liposomes, polymer carriers such as microspheres, hydrogels, polymeric NPs, dendrimers, and nanowires, metallic NPs (e.g., Au, Ag, Ti), carbon nanodevices (e.g., nanotubes, nanodiamonds (NDs), graphene), and inorganic particles (e.g., silica particles) [9,10]. There are various drug delivery systems available for administering drugs to the targeted area of action. Each type of material possesses its own advantages and disadvantages, and the selection of a particular method should be based on individual clinical needs and considerations. Nano-drug delivery systems heavily rely on the utilization of nanoparticles, which are categorized as inorganic materials, organic small molecules, and polymers. The physicochemical properties are altered by changing the chemical composition, structure, size, and shape of the surface. The main types of distributions are shown in Figure 1. Additionally, Table 1 summarizes the advantages and disadvantages of different nanodelivery carriers. This comprehensively overviews the various distribution methods and their associated benefits and drawbacks.

Gold nanoparticles (AuNPs) have gained attention in the field of drug delivery due to their superior properties compared to liposomes, polymers, dendrimers, and metal nanoparticles [25]. These properties include good biocompatibility, a large specific surface area, surface modifiability, and excellent stability and dispersion characteristics [26]. In addition to their effectiveness in delivering various drugs, such as proteins, peptides, nucleic acids, and macromolecules, AuNPs can also stabilize and enhance the efficacy of other drug delivery carriers [4]. AuNPs have firm local surface plasmonic resonance (LSPR), high X-ray radiation absorption capacity, and photothermal conversion performance. These characteristics make them attractive for biosensors, catalysis, and tumor therapy applications, including photothermal and radiation therapy. The potential of AuNPs in these areas is promising [27,28,29]. Additionally, such modifications enable the efficient delivery of drugs to specific targets, thereby enhancing the application of gold nanoparticles in drug delivery [19]. However, despite ongoing developments, inorganic nanomaterials are still not available for clinical use. One of the primary reasons for this is their potential toxicity in the human body. Therefore, it is crucial to focus on modifying and improving these materials. Fortunately, advancements in the field have made it possible to minimize the toxicity they produce through surface modification and the design of biocompatible coatings.

Polysaccharides are a type of carbohydrate composed of monosaccharides linked by glycosidic bonds. They are abundant in nature, making them a readily available and cost-effective resource. Furthermore, due to their chemical structure, polysaccharides can be easily modified to suit specific applications [30]. In addition to their versatility, polysaccharides exhibit a wide range of biological activities. These include antitumor properties, which can help inhibit the growth of cancer cells [31].

Furthermore, polysaccharides possess antioxidation capabilities, contributing to their ability to combat oxidative stress and reduce the risk of various diseases [32]. They exhibit antibacterial effects, enabling them to fight harmful bacteria and protect against infections [33]. Moreover, polysaccharides are known for their hypoglycemic properties, which can help regulate blood sugar levels and support diabetes management. Polysaccharide-based nanocarriers are the focus of current research [34]. The combination of polysaccharides and nanocarriers improves the biological activity of polysaccharides [32] and enhances the biocompatibility of nanocarriers. This increased biocompatibility allows cells to accept the nanocarriers readily and facilitates the efficient delivery of drugs to the desired target location. Meanwhile, polysaccharide nanocarriers enhance drug-loading activity by gradually releasing the loaded medicines [35]. Additionally, gold nanoparticles have unique properties that enable drug release in specific locations, including pH response, temperature response, or photothermal response.

## 2. Biological Activity of Polysaccharides

**Figure 2:**
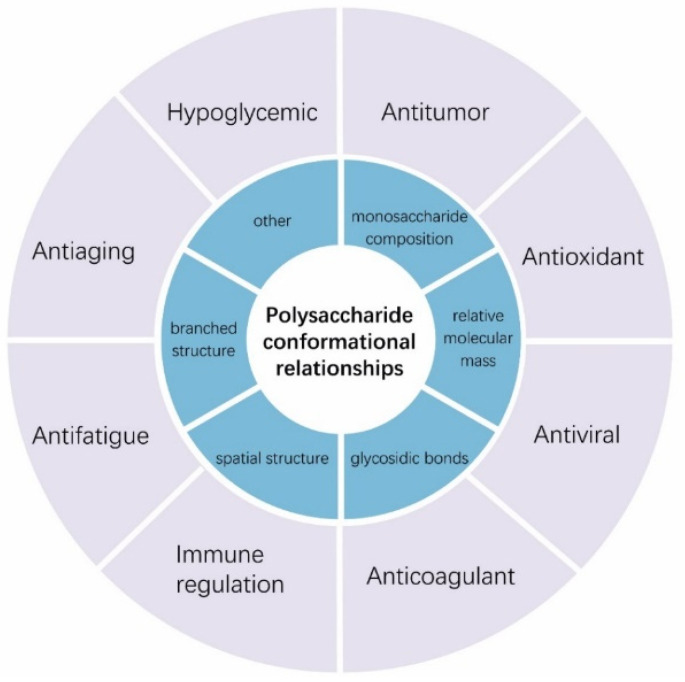
Constitutive relationship of polysaccharides. The image was prepared by Figdraw 2.0.

Polysaccharides, which can be found in abundance in plants, animals, bacteria, and microorganisms, are a prominent type of natural polymers. They possess remarkable immune enhancement properties, promoting improvements in humoral, cellular, and mucosal immune responses. Moreover, these complex molecules have exhibited therapeutic potential in treating diverse diseases, including tumors, infections, and autoimmune disorders [36]. Numerous studies have extensively investigated the various biological activities of polysaccharides, with the primary emphasis placed on immune regulation [37], antitumor [38], antivirus [39], anti-coagulation [40], and antioxidation [32]. The biological activity of polysaccharides is intricately linked to several factors, including monosaccharide composition, glycoside bond type, spatial structure, molecular weight, and branched chain structure [41]. Various factors affecting polysaccharide activity of polysaccharides have been investigated in recent years (Figure 2).

Numerous studies have shown that polysaccharides can interact directly or indirectly with immune cells, which in turn prompts various cellular or molecular reactions. These reactions activate the immune system and ultimately result in immunomodulatory effects. Furthermore, the immune system can recognize and target a wide range of antigens and harmful substances produced by tumor cells in the body. This recognition leads to the elimination of these malignant cells. Therefore, it is highly likely that polysaccharides with immunomodulatory properties may be promising and potent antitumor drugs [42]. Gao et al. isolated SPPs with antitumor and immune-enhancing activities from Sargassum, which could enhance the proliferation of immune cells and increase the expression of serum cytokines, and further analysis showed that it could significantly induce apoptosis while inhibiting the normal physiological process of cancer cells, suggesting that SPPs, polysaccharides isolated from the natural polysaccharide Sargassum, are involved in immune-regulatory effects in various ways and can be used as potential bioactive components of antitumor drugs with immune-enhancing activities [43]. Further research and analysis of the antitumor and immune-enhancing activities and structural features of SPPs are still needed, and secondly, according to the antitumor mechanism of the SPP-0.7 fraction pointed out by the authors in the article, more experiments need to be conducted in the future for validation. This is the first study of the antitumor and immune-enhancing activity of SPPs, which could contribute to the further development of this traditional Chinese herb and seaweed, but in the meantime, further analysis of its structure is still needed. Polysaccharides from Chinese herbs have been found to induce apoptosis in various tumor cells, both in vivo and in vitro. For example, Dendrobium polysaccharides, Astragalus polysaccharides, Lycium barbarum polysaccharides, and panax notoginseng polysaccharides have shown this effect [44,45,46,47]. Polysaccharides from food sources, such as coriander polysaccharides, pomegranate polysaccharides, chestnut polysaccharides, and papaya polysaccharides, also demonstrate antitumor effects [48,49,50,51].

The relative molecular mass distribution of polysaccharides, the composition of sugar groups, the type of glycosidic bonding and the way adjacent glycosidic bonds are connected, the presence or absence of branched chains as well as their position and length, and the non-sugar modifications may have a significant effect on their activity, as shown in Figure 2. Astragalus polysaccharides (APSs) with different Mw values stimulate the proliferation of macrophage-absorbing neutrophil erythrocytes and NK cells to varying degrees, which in turn modulates the intensity of nonspecific immune responses [52]. Different structures and modifications of polysaccharides enable different polysaccharides to have various biological activities, such as antitumor and antioxidant. Non-sugar modifications such as phosphorylation and sulfation also have different degrees of influence on the activity of polysaccharides. Phosphorylation of polysaccharides is a standard method to reduce natural polysaccharides’ high viscosity and increase their water solubility and bioactivity. The negatively charged phosphate groups of phosphorylated polysaccharides can bind with receptors on the surface of immune cells and activate the immune response, resulting in antitumor activity. At the same time, phosphorylated polysaccharides have a higher scavenging capacity for DPPH and superoxide anion [53]. The electrostatic repulsion and spatial resistance of polysaccharides modified by sulfate changed dramatically, which increased the flexibility of polysaccharide chains and improved water solubility, and the sulfated modified yam polysaccharide (S-CYP) could activate macrophages through the MAPK signaling pathway, which showed better immunomodulatory effects than CYP [54].

Meanwhile, polysaccharides that are directly extracted or subjected to sulfation modification have the potential to exhibit improved antiviral activity [55]. Sulfated xymannan, xylan, pectin, fucoidan, dexosan, glucoarabinoglycan, and arabinoglycan are polysaccharides that have been shown to possess antiviral and immunomodulatory activities [56]. Polysaccharides play a crucial antiviral role through various mechanisms, such as binding to the virus’ surface via charge interaction. This binding effectively blocks virus transmission. Additionally, polysaccharides exert their antiviral effect by directly inhibiting viral transcription and replication in host cells. Yermak et al. demonstrated that carrageenan isolated from the Japanese sea red alga Tichocarpus crinitus has a complex structure containing polysaccharides that can inhibit virus-infected cells and that a particular enzyme was used to obtain a low molecular derivative of carrageenan, oligosaccharide, which could be helpful in the development of new drugs against viral infections. Oligosaccharides, low molecular products of carrageenan, were obtained with the help of a particular enzyme. Carrageenan and its oligosaccharides can significantly inhibit immunodeficiency viruses and retroviruses (10-fold and 7-fold inhibition, respectively), which will be helpful for the development of new drugs against viral infections [57]. However, further ex vivo and in vivo studies are needed to evaluate the results.

Furthermore, they indirectly activate innate immunity, further enhancing their antiviral properties. Coriolus versicolor’s polysaccharide peptide (PSP) has been shown to have immunomodulatory properties, activating the innate immune response through toll-like receptor 4 (TLR4), confirming the potential use of PSP as an anti-HIV drug, but studies in a larger number of infected subjects are still needed [58]. 

Polysaccharides are antioxidant in three main ways: first, through the modulation of antioxidant enzyme activities via the Nrf2/ARE pathway; second, by removing free radicals; and third, by antagonizing nitric oxide. The expression of CAT, SOD, and GSH-Px, the main antioxidant enzymes in the human body, is regulated by Nrf2. When oxidative stress occurs in the human body, Nrf2 is released from Keap1, enters the nucleus, and interacts with the antioxidant response element (ARE) [59]. Lentinan (LNT) activated the Nrf2 pathway and inhibited hepatocyte oxidative stress [60]. Polysaccharides can induce the expression of antioxidant genes or proteins and improve antioxidant effects through the Nrf2/ARE pathway. When the body’s average free radical production and elimination is imbalanced due to various external factors, polysaccharides effectively scavenge free radicals and produce antioxidant effects by regulating and enhancing the activity of oxidative enzymes. Elaeagnus umbellata polysaccharides have scavenging or inhibitory effects on hydroxyl radicals and superoxide anion radicals and can be used as natural antioxidants. It was also found that polysaccharides with different molecular weights have different degrees of antioxidant activity, and that low-molecular-weight polysaccharides may have more significant antioxidant activity due to the fact that they have more reducing hydroxyl terminals [61]. Finally, polysaccharides can improve antioxidant capacity by inhibiting inducible NOS (iNOS) and reducing NO concentration. Angelica sinensis polysaccharide (ASP) inhibits H_2_O_2_-mediated oxidative stress injury in human chondrocytes by down-regulating iNOS, SOD, and CAT for osteoarthritis (OA) treatment [62].

Although polysaccharides have a variety of biological activities in experimental studies, they have not yet been directly and widely used in clinical studies due to the following points: firstly, the complex way in which polysaccharides interact with immune cells, and their effects can vary depending on their structure, concentration, and source; secondly, different sources of polysaccharides may have different compositions and biological activities, which makes it difficult to standardize them for use in clinical studies; and finally, although polysaccharides have potential immunomodulatory effects, there may be safety issues with the use of polysaccharides in clinical studies. The safety of polysaccharides, including potential adverse reactions and interactions with other drugs, must be thoroughly investigated before using them in clinical trials. Despite these challenges, there is ongoing research into the potential therapeutic applications of polysaccharides, and with continued advancements in scientific understanding and technology, it is possible that polysaccharides may eventually be used more directly in clinical research.

## 3. Preparation of Polysaccharide Gold Nanoparticles

AuNPs have several advantages, such as inertness, non-toxicity, good biocompatibility, a large specific surface area, and ease of modification, making them widely used in drug delivery. AuNPs constitute a diverse family of nanoparticles, which includes nanoclusters, nanocages, nanowires, nanorods, nanotubes, nanostars, nanocombs, nanoribbons, and nanoshells [63]. Two main strategies for synthesizing gold nanoscale particles are top-down and bottom-up. Top-down synthesis involves techniques such as laser ablation, ion sputtering, ultraviolet and infrared radiation, etc. On the other hand, bottom-up synthesis methods involve the reduction of Au(iii) to Au and can be categorized into chemical and biosynthetic processes [64]. Chemical reduction involves the reduction of gold ions using a reducing agent such as sodium borohydride or citrate. This is a relatively simple and widely used method for preparing gold nanoparticles. However, control over the size and shape of the resulting particles can be quite limited. The use of physical methods for the synthesis of AuNPs allows for precise control of the size and shape of the nanoparticles, minimal chemical contamination, and the ability to produce AuNPs with application-specific properties. However, it is important to carefully optimize the process parameters in order to obtain the desired nanoparticle properties. Biosynthesis is environmentally friendly and usually allows for better control over the size and shape of the particles. However, optimizing and controlling the synthesis process can be more challenging. Several syntheses of gold nanoscale are summarized in Table 2.

**Figure 3:**
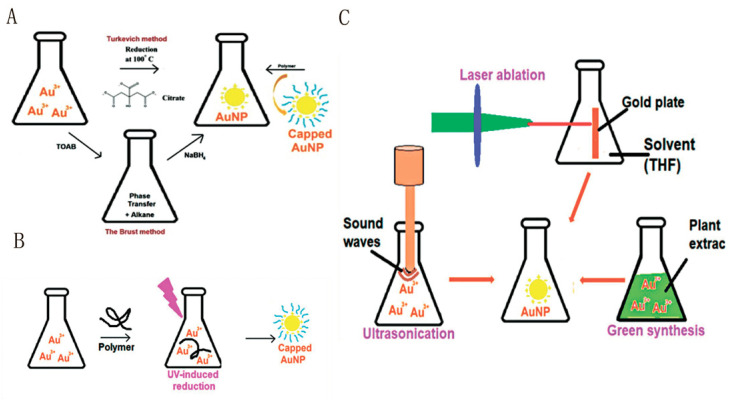
Synthesis of gold nanoparticles. (**A**): Turkevich–Frens method. (**B**): UV synthesis of gold nanoparticles. (**C**): Ultrasonic, laser ablation, and plant extract-based AuNPs. Reproduced with permission from Ref. [64].

The main physical methods for synthesizing AuNPs include physical fragmentation, evaporation condensation, plasma precipitation, and sputtering. In addition, gold nanoscale can be synthesized using UV-induced photochemistry, laser ablation, and ultrasound-assisted synthesis. Figure 3B,C present synthetic reduction schemes for AuNPs using UV-induced, ultrasonic, laser ablation, and plant extract-based AuNPs, respectively. Wan et al. utilized the sputtering method to load AuNPs onto the surfaces of carbon nanocoils. This technique resulted in the creation of a visible-light-driven photocatalyst [65]. Rehab et al. conducted a study where they successfully synthesized chitosan/gold nanocomposites (Cs/Au) through γ-irradiation. The authors’ findings demonstrated that these Cs/Au nanocomposites exhibit remarkable antimicrobial and anti-biofilm activity. Additionally, they showed the ability to inhibit the growth of cancer cells. These significant results underscore the immense potential of Cs/Au nanocomposites in the biomedical and pharmaceutical fields [66]. 

Chemical methods are widely used for the synthesis of AuNPs. Turkevich–Frens first used the most common and long-established method for preparing AuNPs in 1951(Figure 3A) [67]. This method utilizes relatively green reagents, and the synthesis process is reasonably simple. It involves the formation of 10–20 nm AuNPs by reducing chloroauric acid in boiling water using sodium citrate as a reducing agent and stabilizer. However, the concentration of NPs was relatively low. Researchers have been studying the impact of different factors, including reagent concentration, order of reagent addition, the addition of other ions, pH, temperature, time, and synthesis method on the size of gold nanoscale using Turkevich’s method. Additionally, considerable effort has been dedicated to understanding the nucleation mechanism and gold nanoscale growth [68]. Ojea-Jiménez et al. [69] pointed out that the hydroxyl group in the citrate is in the α-position with a carboxylate, which allows the formation of a cyclic pentameric complex in the construction of intermediates, i.e., cyclic five-membered complexes, in the rate-determining step [69]. The Brust–Schiffrin (BBS) method is a highly effective technique for synthesizing thiol-functionalized AuNPs, known for their stability. The BBS method involves the transfer of AuCl_4_ from the water phase to toluene phase, reducing Au(iii) to Au metal and stabilizing the generated AuNPs. The AuNPs synthesized by this method usually have a small size (2–6 nm) and narrow size distribution, and the obtained AuNPs can be easily functionalized and modified by subsequent ligand exchange, while it can synthesize many AuNPs [70]. Seed-mediated synthesis strategies, in which small gold nanoparticle precursors are added to a growth solution to initiate heterogeneous nucleation, are among the most prevalent, simple, and productive methodologies for generating well-defined colloidal anisotropic nanostructures [71]. 

**Figure 4:**
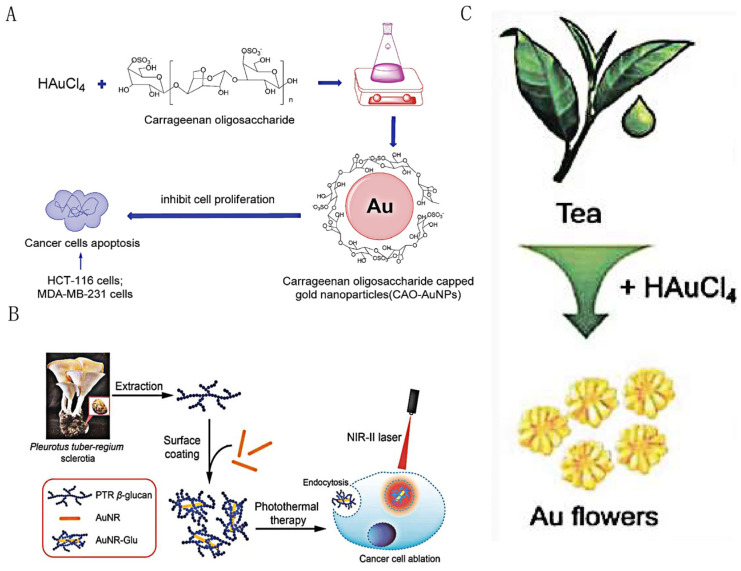
Green synthesis method for gold nanoscale. (**A**): CAO-reduced Au^3+^ ions and capped AuNPs. Reproduced with permission from Ref. [74]. (**B**): Mushroom β-glucan-modified AuNRs under NIR for photothermal therapy of cancer. Reproduced with permission from Ref. [75]. Copyright 2017, American Chemical Society. (**C**): Tea-assisted synthesis of gold nanoflowers. Reproduced with permission from Ref. [76]. Copyright 2022, Tsinghua University Press.

Biosynthesis of AuNPs is considered a safe, dynamic, and energy-efficient method (Figure 4). Compared to other methods, AuNPs produced through biological means exhibit enhanced stability. This stability is attributed to the presence of metabolites, including proteins, fatty acids, sugars, enzymes, and phenolic compounds, produced by various organisms. These metabolites have a dual role in both the formation of AuNPs and the maintenance of their stability. Cyanobacteria, for instance, can synthesize AuNPs both intracellularly and extracellularly. It is postulated that the reduction of Au ions by cyanobacteria during biosynthesis leads to the formation of AuNPs. Moreover, extensive studies have demonstrated that the AuNPs produced by cyanobacteria do not exhibit toxicity towards normal and cancerous human cells [49,72,73]. CAO extracted from red algae has a dual role as a reducing and stabilizing agent and can be used to synthesize AuNPs. Carrageenan oligosaccharide (CAO) reduced Au3+ions and capped gold nanoparticles (AuNPs) and, subsequently, the cytotoxicity of CAO-AuNPs on cancer cells (HCT-116 cells and MDA-MB-231 cells) (Figure 4A). This study provides a reference for green preparation of AuNPs from marine carbohydrates [74]. 

Fungi contain enzymes and proteins as reducing agents, so they can always be used to synthesize metal nanoparticles from their salts, but care needs to be taken with their pathogenicity in experiments. These AuNPs can be synthesized directly on the surface of mycelium when fungus is present [18]. Zhang et al. successfully biosynthesized gold nanoparticles (AuNPs) and platinum nanoparticles (PtNPs) using yeast microcapsules. These nanoparticles were employed to stimulate the immune response and improve the efficacy of antitumor treatment. This approach was supplemented with chemodynamic therapy (CDT) and photothermal therapy (PTT), leading to a synergistic effect. Nevertheless, the therapeutic impact and range of tumor treatment could be further improved, and this experimental protocol requires further optimization [77]. 

A wide range of gold nanomaterials with varying sizes and shapes, such as gold nanospheres, gold nanorods (AuNRs), gold nanoshells, gold nanocages, gold nanoclusters (AuNCs), and nanostars, have been fabricated [78]. Different morphologies dictate distinct properties, and AuNPs with anisotropic shapes (e.g., AuNRs and gold nanostars) or hollow structures (e.g., gold nanoshells and gold nanocages) can exhibit LSPR peaks in the near-infrared (NIR) region [79]. AuNRs are rod-like gold nanomaterials that are commonly utilized in light-induced photothermal (photodynamic) therapy [80], gold nanocages, characterized by hollow interiors and porous walls, are considered more preferable drug delivery vehicles [81]; gold nanostars possess three distinct surface curvatures, making them exceptionally valuable for biological sensing, imaging, and localized chemical manipulations [82], gold nanoclusters, are ultra small in size (typically less than 3 nm in diameter) and possess unique fluorescent properties. These nanoclusters exhibit remarkable tumor accumulation and efficient renal clearance properties, rendering them agile probes for in vivo imaging [83]. The longitudinal surface plasmon resonance (LSPR) peaks of AuNR nanorods are absorbed in the NIR region, causing them to produce NIR light-induced photothermal effects that kill tumor cells and thus are used for tumor PTT [84,85]. However, such nanomaterials are less stable under physiological conditions and tend to aggregate and form precipitates. Isolation and purification of homogeneous polysaccharide BCP50-2 from *Belamcanda chinensis* (L.) DC can be used to stabilize gold AuNRs and prepare to generate polysaccharide-AuNPs with good stability and high efficiency of photothermal conversion, which is an excellent guide for tumor therapy and has a better prospect of application in the treatment of hepatocellular carcinoma [86]. 

Gold nanoparticles (AuNPs) have unique properties that make them potentially useful for biomedical applications, including drug delivery, imaging, and therapy. However, there are several reasons why AuNPs are not yet widely used in clinical applications. First, there is the issue of safety; while AuNPs are generally considered biocompatible, their long-term effects on human health and their potential to accumulate in the body have yet to be thoroughly investigated. Secondly, the lack of standardized production does not ensure that the gold nanoparticles produced have consistent particle sizes, surface properties, etc. Again, there is limited understanding of its distribution, metabolism, and clearance in the body. Finally, there is the challenge of scaling up the production of AuNPs. Although the technology for small-scale synthesis of AuNPs has matured, there are still a number of technical challenges that need to be resolved in order to scale up the production to meet the needs of clinical use.

## 4. Application of Polysaccharide-AuNPs

**Figure 5:**
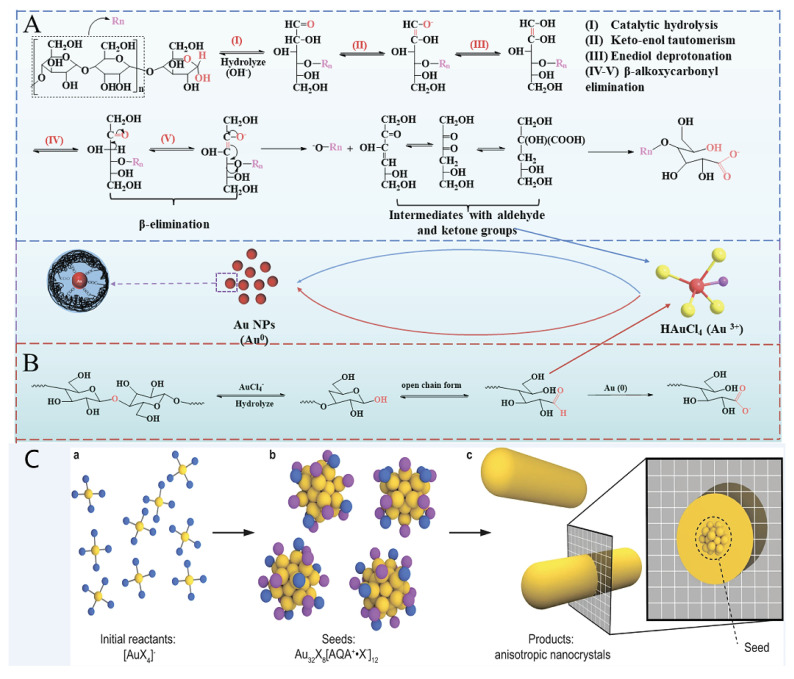
(**A**): Chloroauric acid–mediated glycosidic bond–breaking synthesized gold nanoparticles. (**B**): Base–mediated glycosidic bond–breaking synthesized gold nanoparticles. Reproduced with permission from Ref. [88]. (**C**): Gold nanosynthesis with halogenated gold salts (**a**) to generate seeds (**b**), which in turn form well-defined shapes (**c**). Reproduced with permission from Ref. [71].

Polysaccharides have immunomodulatory functions; however, the efficacy of polysaccharides for cancer therapy remains poor. One possible reason is their low prescribed dose and rapid clearance in the clinical setting. One solution to this issue is combining polysaccharides with nanomaterials, which can enhance the immunostimulatory activity of polysaccharides and produce a more substantial tumor therapy effect. In particular, preparing AuNPs using plant extracts offers several advantages over synthetic approaches. This method provides a more accessible and cheaper route to industrial scale-up and has a higher safety profile for humans [87]. The chemical groups present in polysaccharides give them the ability to act as reducing or stabilizing agents in preparing AuNPs. The mechanisms involved in synthesizing AuNPs from polysaccharides can be classified into three categories, depending on the specific type of polysaccharides and the reaction conditions. These categories include glycosidic bond breaking, hydroxyl group reduction, and amino group reduction (Figure 5) [88].

Zhang et al. conducted a study in which they synergistically combined immunologically active Ganoderma lucidum polysaccharides with gold nanomaterials. This combination proved effective in inducing the maturation of DCs, stimulating the proliferation of CD4+ and CD8+ T cells, and inhibiting the growth and metastasis of tumor cells. Notably, the researchers discovered that the addition of doxorubicin to the combined treatment further enhanced its effectiveness in treating tumors, and the combination of polysaccharides and nanomaterials can achieve the same therapeutic effect while reducing the dose of chemotherapeutic drugs and polysaccharides [89]. Polysaccharides can be utilized as reducing and stabilizing agents to synthesize polysaccharide-AuNPs, which can efficiently safeguard orally administered insulin from degradation. This is because polysaccharides remain undigested in the gastrointestinal tract, making them suitable for treating diabetes mellitus. Yogita et al. confirmed the above using apple polysaccharide-AuNPs, but the mechanism and pharmacokinetics still need further study [90]. 

Polysaccharides alone and AuNPs alone have limitations in disease treatment. However, when polysaccharides are combined with AuNPs, the resulting polysaccharide-AuNPs can offer a more promising approach. By combining the targeting ability of gold nanoparticles with the biological activity of polysaccharides, polysaccharide-AuNPs exhibit enhanced antitumor, immunocompetence, and antiviral effects. They also possess the targeting, photothermal effects, and carrier effects of AuNPs. As a result, the synergistic integration of both components allows polysaccharide-AuNPs to better fulfill their role in disease treatment.

The mechanisms of preparation of polysaccharide-based AuNPs is categorized into three types: glycosidic bond disruption via Au(III) or base-mediated generation of highly reduced intermediates, reduction of free hydroxyl groups, and reduction of free amino groups. Polysaccharides are degraded under base-mediated conditions to intermediates containing aldehydes or α-hydroxyketones, which are subsequently oxidized to carboxylation by-products to maintain the stability of AuNPs (Figure 5A). The Au (III) ion promotes intramolecular or intermolecular nucleophilic attack, leading to the protonation of the oxygen atom on the glycoside, triggering a break in the glycosidic bond. The O-5 and C-1 bonds within the inner ring then undergo heterogeneous decomposition, exposing highly reduced free aldehyde or ketone groups that facilitate the Au (III) ion to Au (0) atoms (Figure 5B). The seed growth process begins by nucleating seed particles (b) through the rapid reduction of the gold halide salt (a). These seed particles then serve as non-homogeneous nucleation sites in a subsequent reaction, leading to the controlled growth of particles with a well-defined shape (c) (Figure 5C).

Polysaccharide-AuNPs exhibit many distinctive bioactivities, marked antimicrobial properties, and versatility as indicators and biosensors. Vijaya Kumar et al. conducted a study to evaluate the in vitro proliferative activity, antimicrobial activity, and antifungal activity of longan polysaccharide-AuNPs. The results of this study demonstrated that the prepared longan polysaccharide-AuNPs exhibited excellent in vitro proliferative activity and in vitro antimicrobial and antifungal activity [87]. The design of AuNPs-based biosensors follows a fundamental principle by functionalizing or covering the AuNPs with thiolated biomolecules. This modification significantly changes the light absorption of the AuNPs when they come into contact with complementary biomolecules [91]. Depending on the color change, the AuNPs obtained by Wang et al. using marine algal polysaccharides can serve as convenient, reliable, safe, and inexpensive time–temperature indicators for monitoring the quality and safety of perishable bioproducts. However, their application in practical production still needs to be studied for improvement [92]. 

Meanwhile polysaccharide gold nanosystems have been widely used to prepare various biodetectors due to their convenience, speed, and sensitivity. In 1997, Zehbe et al. made a significant advancement in using nanomaterials for virus detection by combining AuNPs with silver staining to detect HPV in cervical cancer cells. This pioneering work marked an important turning point in the field [93]. The attachment of hyaluronic acid (HA)-functionalized AuNPs/polyetherimide (PEI)-stabilized/rhodamine B (RhB) (AuNPs/PEI/RhB-HA) materials to CD44 receptor overexpressing MCF-7 cancer cells has been investigated as a means of developing specific probes for in vivo cancer diagnosis. However, the method was less biocompatible, and the effects were not explored [94]. Salma et al. synthesized chitosan-capped AuNPs for simple, rapid, and green detection of Mycobacterium tuberculosis (MTB) by PCR, but the protocol has a small scope of application because free chitosan in AuNPs preparation affects the results of the experiments and there is difficulty in performing the assay on multiple samples [95].

The abuse of antibiotics worldwide has led to bacterial infections becoming a significant threat. Extensive research has been conducted on various gold-based nanostructures as potential antibacterial agents. These nanostructures exhibit exceptional chemical and physical characteristics, making them promising tools to fight against bacterial infections [96]. In their research, Cardenas et al. conducted a study to investigate and assess the antimicrobial properties of metal AuNPs. Specifically, they examined the effects of Au, Ag, and Cu nanoparticles when combined with HA. The objective of this study was to determine the antimicrobial efficacy of the combination of these metal AuNPs with HA. The authors’ findings indicate that the collective use of metal AuNPs (Au, Ag, Cu) with HA significantly increases in antimicrobial activity, surpassing the effects observed with individual metal nanoparticles or HA alone [97]. 

Chitosan (CH) is a natural polysaccharide derived from chitin, a substance found in the shells of crustaceans. It has been widely studied for its antimicrobial properties and has shown great potential in various applications, including drug delivery and wound healing. Both CH and silver AuNPs have demonstrated potent antibacterial activity. To further improve the stability and antibacterial efficacy against multidrug-resistant pathogenic bacteria and reduce toxicity in humans, the conjugation of the biopolymer CH with silver or gold nanoparticles offers a promising approach [98]. Cs-AuNPs have been demonstrated as efficacious bactericidal materials that circumvent damage to human cells. Including a positively charged CH amplifies its interactions with bacteria, thus allowing the positively charged AuNPs to disrupt the anionic bacterial cell membrane efficiently. As a result, CH enhances the biocompatibility and antibacterial activity of AuNPs, and charge density is an essential parameter for antimicrobial activity, and only chitosan that meets specific conditions can demonstrate good antimicrobial activity in the region [99]. The study conducted by Inbaraj et al. focused on synthesizing gold nanoparticles (AuNPs) using CH, glycol chitosan (GC), and poly(γ-glutamic acid) and investigating their antimicrobial properties. Their findings provided compelling evidence to demonstrate the remarkable antimicrobial properties of these CH-coated AuNPs, but further elucidation of their antimicrobial mechanism is still needed [100]. 

Tumorigenesis refers to the process by which normal cells transform into cancer cells. It is a multistage and progressive process involving genetic and epigenetic changes in cells. During tumorigenesis, these altered genes can confer new capabilities to the affected cells, such as uncontrolled proliferation, evasion of cell death, evasion of the immune system, and the ability to invade nearby tissues and spread to distant sites (metastasis). Each of these capabilities evolves over time, leading to a tumor’s subsequent formation and progression [101]. Biofouling refers to the undesired adsorption of cells, proteins, or intracellular and extracellular biomolecules on the surface of metal nanocomplexes. In this context, HA is a bioactive, naturally occurring mucopolysaccharide. HA is hydrophilic and polyanionic in physiological environments, exhibiting excellent antifouling properties. A noteworthy finding is that HA-thiol (HA-SH) (MW 10 kDa) of AuNPs displayed higher peritumor distribution in lung cancer compared to gold nanoparticles alone and lower distribution in normal organs. This observation offers promising possibilities for utilizing nanomaterials in tumor diagnosis and therapy [102]. Manivasagan et al. have designed and developed a new multifunctional nanocarrier known as anti-epidermal growth factor receptor antibody-conjugated and paclitaxel loaded-thiol chitosan-layered gold nanoshells (anti-EGFR-PTX-TCS-GNSs). This new nanocarrier has been shown to kill tumor cells almost wholly under laser irradiation. Moreover, it possesses a solid ability to visualize tumors. Consequently, this nanocarrier presents a novel strategy for future tumor diagnosis and treatment, offering a new approach in the field [103]. Pholiota adiposa (PA) is a widely used edible and medicinal mushroom in traditional Chinese medicine. This mushroom is known for containing a polysaccharide (PAP-1a), considered its primary physiologically active component. In a study conducted by Yang et al., it was found that by synthesizing AuNPs using PAP-1a, there was a significant improvement in immune regulation and antitumor effect in vitro and in vivo compared to using PAP-1a alone. This discovery demonstrates the potential of PAP-AuNPs as a novel nanomedicine for hepatic carcinoma [104].

Central nervous system malignancies are characterized by the lowest survival and highest morbidity rates. This is mainly attributed to the presence of the blood–brain barrier, which restricts the accessibility of drugs to the tumor microenvironment. To overcome this limitation, a potential solution is the use of ultrasmall spherical AuNPs that are capped by galactoyl dextran PST001. These AuNPs have the ability to target antitumor and tumor necrosis factor-associated apoptosis-inducing ligands, and crucially, they can traverse the blood–brain barrier to enhance the distribution of the drug doxorubicin (DOX) in the brain. By employing this approach, DOX exhibits a longer retention time in plasma, reduced distribution in vital organs, enhanced distribution in the brain, and significant inhibition of glioma tumor growth. It is worth highlighting that the improved distribution and inhibition of tumor growth are of utmost importance for the palliative treatment of drug-resistant brain tumors, but no studies have been made on the therapeutic effects of this drug carrier in vivo [105].

## 5. Research on Polysaccharide-AuNPs as Drug Delivery Carriers

**Figure 6:**
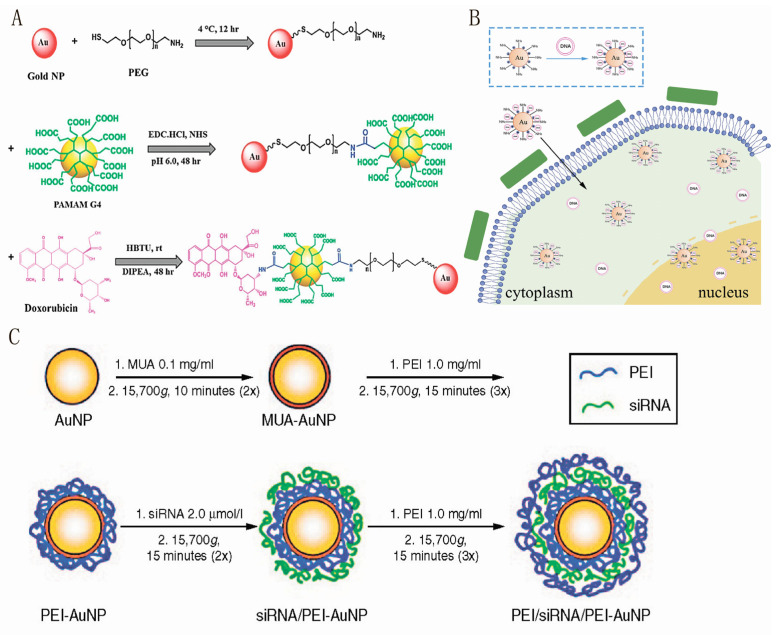
(**A**): PEG-modified gold nanoparticles used to deliver DOX. Reproduced with permission from Ref. [127]. (**B**): Schematic representation of AuNPs-PEI-pDNA complexes entering and expressing in cells. Reproduced with permission from Ref. [128]. (**C**): Positively charged PEIs and coated mercaptoundecanoic acid MUA-stabilized AuNPs delivering siRNAs. Reproduced with permission from Ref. [123].

Drugs can be directly bound to the surface of AuNPs through covalent and non-covalent interactions (Figure 6). When employing polysaccharide-AuNPs for drug delivery, medications can be attached to the nanomaterials via several methods, including physical coating, chemical grafting, or electro-attraction. The diverse binding modes of polysaccharide-AuNPs enable the delivery of a wide array of drugs, encompassing small molecule drugs, nucleic acids, proteins, and peptides [106]. In addition, the surface of AuNPs can be modified to facilitate drug encapsulation and delivery. Depending on the properties of the drug, various modifications can be performed on polysaccharide-AuNPs to meet the needs of delivering different types of drugs. Furthermore, the binding of proteins or peptides to AuNPs can occur through electrostatic interactions. DNA is negatively charged due to the phosphate backbone. However, most AuNPs, commonly used in academic research, also have a negative charge on their surface. This poses a challenge when attempting to combine nucleic acids with AuNPs using electrostatic adsorption. As a result, modifying the AuNPs or the nucleic acids is necessary for successful integration. The most commonly employed method involves changing the nucleic acids through sulfhydryl modification. It has been demonstrated that AuNPs with diameters ranging from 1 to 100 nm can successfully accommodate a significant quantity of sulfhydryl-modified oligonucleotides. Consequently, this technique offers a valuable strategy for incorporating nucleic acids into AuNPs [107]. To enhance the delivery of various payloads, it is essential to functionalize AuNPs. This can be achieved through multiple methods, including glycation, peptide, and amino acid binding, or by functionalizing them with oligonucleotides. The functionalization process enables AuNPs to transport and deliver a diverse range of payloads effectively [108].

Modification of nanomaterials can improve their stability and targeting capabilities and broaden the range of nanomaterial applications. The function of gold nanometers is influenced by the material bound to its surface and the nanometer’s size and molecular weight. Polyethylene glycol (PEG) modification is a widely used method for nanomaterials. PEG, being highly biocompatible and hydrophilic, offers several advantages. Firstly, it improves the colloidal stability of nanoparticles, promoting their long-term dispersion in a liquid medium. Secondly, using PEG reduces the immunogenicity of nanoparticles, minimizing the potential immune response upon their introduction into a biological system [109]. Finally, PEG modification prolongs the circulation time of nanoparticles in vivo, enhancing their therapeutic effectiveness. PEG-modified AuNRs exhibit a favorable photothermal effect and demonstrate exceptional efficacy in ablating cervical cancer cells using NIR irradiation [110]. 

Peptide-modified gold nanoparticles have been widely recognized for their bioactivity in various fields, such as drug delivery, cancer therapy, targeting, and biosensors. The complex’s selectivity can be improved by adjusting the density or orientation of the coupled peptide [111]. For instance, Albertini et al. utilized arginine-glycine-aspartate-like peptide-modified gold nanoparticles in their study of cancer diagnosis and therapy, which showed higher accumulation in subcutaneous tumors than unmodified gold nanoparticles, and similar results were obtained in an intracranial tumor model, which demonstrated better penetration of the blood–brain barrier, which initially proves its application in a wide range of tumors. However, more in-depth characterization still needs to be conducted [112]. The efficacy of gold nanoparticles modified with cell-penetrating peptides (CPPs) will be evaluated in terms of their ability to target cancer cells selectively and potentially enhance normal fibroblast function [113]. In a recent study, Guo et al. investigated the role of erythrocyte membranes and antibody-modified AuNRs as nanocarriers for improving antitumor effects. Their findings suggest that these nanocarriers may be able to diagnose and treat pancreatic cancer through active targeting [114]. Chen et al. demonstrated superior radiosensitization with albumin-AuNPs compared to AuNPs alone. Additionally, the albumin-AuNPs showed effective tumor accumulation. These findings highlight the potential for relatively greater radiosensitization and antitumor activity while minimizing damage to normal body tissues, but pharmacokinetic studies, validation of tumor models, and investigation of the mechanism of radiosensitization were lacking in this experiment [115]. 

Thioglycolic chitosan-coated gold nanoshells (TC-AuNSs) can be used as antimicrobial agents against a variety of bacteria such as Staphylococcus aureus, Pseudomonas aeruginosa, and Escherichia coli, which destroy antibiotic-resistant pathogens under NIR laser light to efficiently and rapidly kill the bacteria and prevent bacterial regeneration [116]. AuNPs have the capability to adsorb proteins, resulting in the formation of protein-coated AuNPs [117]. These protein-coated AuNPs are commonly employed for the targeted delivery of proteins or polypeptides to specific locations. The adsorption mechanism might be attributed to electrostatic interactions between the negatively charged surface of AuNPs and the proteins’ positively charged amino acid residues, resulting in protein attachment to the AuNPs surface. Another plausible mechanism could involve protein neutrality when the pH approaches its isoelectric point. The protein’s electrostatic adsorption with AuNPs might diminish at pH values near its isoelectric end. However, given the considerable surface tension of the protein and its weakly hydrated state, it becomes more prone to adsorption onto the AuNPs surface. Tian et al. [118] conducted a study in which they loaded a matrix metalloproteinase-2 (MMP2)-responsive M2pep fusion peptide (M-M2pep) onto the surface of hyaluronic acid-modified AuNRs. The researchers found that M-M2pep was released from the AuNRs when exposed to NIR light irradiation. This release of M-M2pep resulted in an enhancement of the immunoreactivity of the tumor microenvironment (TME).

**Figure 7:**
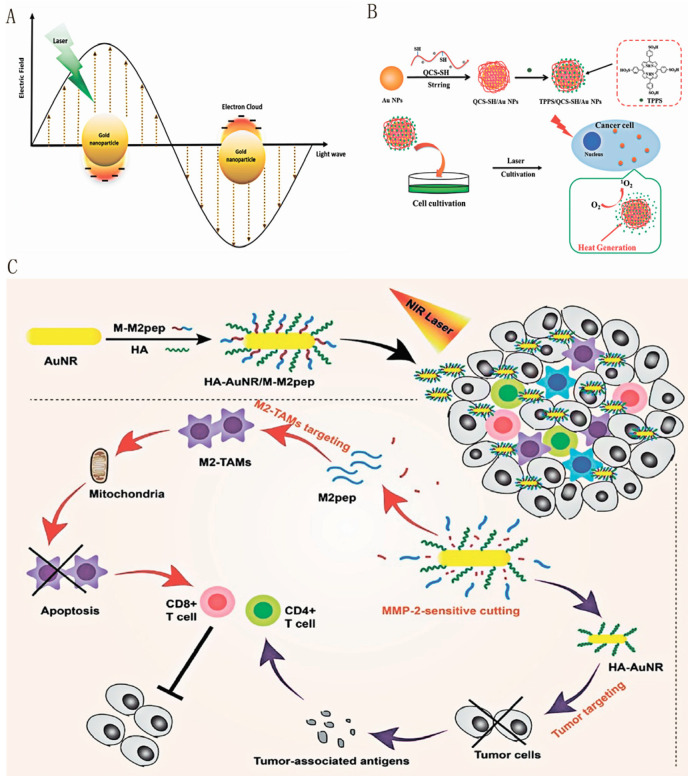
The photothermal effect of gold nanoscale in tumor therapy. (**A**): The LSPR phenomenon on the surface of gold nanoparticles under irradiation at specific light wavelengths. Reproduced with permission from Ref. [140]. (**B**): Illustration for preparing TPPS/QCS-SH/AuNPs and their anticancer properties by PDT/PTT. Reproduced with permission from Ref. [143]. Copyright 2018, American Chemical Society. (**C**): Enhanced photo-immunotherapy by the combination effect of PTT-induced immune activation and M2-TAMs depletion-induced ITME modulation based on HA-AuNRs/M-M2pep. Reproduced with permission from Ref. [118]. 2021 Published by Elsevier B.V.

Additionally, the study demonstrated that HA-AuNRs facilitated precise photothermal therapy (Figure 7C) [118]. Radeepa et al. conducted a study focused on synthesizing AuNPs using Lactobacillus plantarum extracellular polysaccharides (EPS) through green technology. The study was conducted to develop a novel nanocarrier for the delivery of multiple antibiotics to treat multidrug-resistant (MDR) bacterial infections effectively. Experiments demonstrated that the nanocarriers possessed excellent bacterial inhibitory activity, but investigations still need to be carried out in animal models to investigate their optimal antibacterial activity and biodistribution [119].

Polysaccharide-based nanocarriers have emerged as critical players in oral drug delivery. Their molecular structure enhances their binding efficiency and stability with proteins/peptides. This is due to their desirable characteristics, including low immunogenicity, high biocompatibility, and effective lymphatic absorption [120]. Bone morphogenetic protein 2 (BMP-2) is a soluble peptide that can activate cartilage formation. However, its high diffusivity leads to inadequate uptake by target cells, thus limiting its application. Sansanaphongpricha et al. aimed to address this issue by utilizing HA-AuNRs to bind to the peptide. Through this approach, they could effectively enhance the peptide uptake by the cells, ultimately promoting cartilage repair and regeneration [121].

There are electrostatic repulsive forces between nucleic acids and AuNPs, which prevent their combination. However, adding salt can gradually reduce these awful forces, ultimately allowing for the binding of AuNPs and nucleic acids [122]. There are two main ways to bind AuNPs to nucleic acids: (1) using cation-modified gold nanoparticles to bind nucleic acids to gold nanoparticles by electrostatic adsorption. (2) Modification of nucleic acids. Combining nucleic acids and AuNPs using a layer-by-layer deposition method provides a strategy for the controlled release of nucleic acids. Layer-by-layer coating of thioglycolic undecanoic acid-stabilized AuNPs with positively charged PEI and negatively charged siRNA layers yielded much higher cellular uptake of both siRNA/PEI-AuNPs and PEI/siRNA/PEI-AuNPs than that of citrate-stabilized AuNPs (Figure 6C) [123]. DNA was thiolated (-SH) and self-assembled using Au-S bonds to obtain DNA covalently bound functionalized AuNPs [124]. AuNPs have been utilized as carriers for nucleic acid delivery for several years. Initially, this was achieved through electrostatic adsorption of PEI. Subsequently, thiol-capped siRNAs were chemisorbed on the AuNPs [125]. However, research on polysaccharide-AuNPs for nucleic acid drug delivery in oligonucleotides and siRNAs is promising. SiRNAs are an immensely potent therapeutic tool due to their straightforward working mechanisms. They determine target genes by recognizing specific sequences and subsequently regulate their expression. However, the efficient delivery of siRNAs to the target organ has perpetually posed a daunting challenge that demands a solution [126].

The positively charged AuNPs-PEI-pDNA complex can be attached to the cell periphery by electrostatic adsorption and enter the cell wall through the pores. Subsequently, it enters the cytoplasm through endocytosis at the cell membrane and further enters the nucleus. Due to the proton sponge effect of PEI, AuNPs-PEIs release the pDNA they carry to express the corresponding proteins through transcription and translation (Figure 6B) [128]. Au- and PEI-based couplers, modified with HA and DOX, deliver siRNAs while protecting against degradation and promoting their escape from endosomes. This enables a selective treatment for cancers [129]. Fan et al. developed a nanosystem using HA-AuNRs, to which siRNA against B7-H3 (B7-H3si) and glucose oxidase (GOx) were attached. This nanoplatform was designed for efficient low-temperature PTT. The study aimed to inhibit tumor cell proliferation, induce cell apoptosis and achieve effective tumor ablation under mild, low-temperature conditions (42–45 °C) while utilizing RNA interference [130]. The surface modification of AuNPs with HA and low molecular weight PEI enables the efficient transport of siRNA. This modified system serves multiple purposes, including gene silencing, photothermal therapy, and chemotherapy, which are vital in breast cancer treatment [129].

AuNPs can generate localized heat and deliver drugs near cancerous tissues. DOX, commonly used in clinical and research settings as an antitumor drug, has been the subject of numerous studies investigating the impact of polysaccharide-AuNPs on its delivery. Rabiee, N. et al. further stabilized AuNPs with mercapturized poly(ethylene glycol) and then covalently coupled them with poly(amidoamine) G4 dendrimers for efficient and specific delivery of doxorubicin. The gold nanocarriers prepared by this modification method have better colloidal stability and biocompatibility, and at the same time, they can be used as pH-triggered multifunctional nanoplatforms (Figure 6A) [127]. These studies aim to explore the potential of polysaccharide-AuNPs in tumor treatment. A survey by Manivasagan et al. focused on synthesizing AuNPs using fucoidan to deliver the anticancer drug DOX. As demonstrated by in vitro experiments, it was found that modification of fucoidan improved the surface properties of the AuNPs and enhanced the binding and delivery of DOX to the polysaccharide-AuNPs. However, the effects of in vivo administration have not been explored and pharmacokinetics have not been studied. Notably, fucoidan AuNPs have also been used in photoacoustic imaging and as contrast agents [131]. Archana et al. conducted a study in which they synthesized gold nanoparticles (AuNPs) using a polysaccharide obtained from pomegranate peels. The inclusion of galactose in the pomegranate polysaccharide-AuNPs serves as a tumor-homing sequence, enabling targeted delivery of the antitumor drug DOX to the tumor tissues. This polysaccharide-AuNPs system enhances the accumulation of DOX in the tumor tissues while reducing potential damage to normal tissues [132].

In the field of research on antitumor drugs, there has been a growing interest in the efficacy of various compounds, including PTX [133], cephalexin (CPT) [133], irinotecan hydrochloride (CPT-11) [134], and topotecan hydrochloride (TPT) [135]. These drugs have gathered attention due to their potential therapeutic benefits, in addition to DOX. The HA-AuNPs prepared by Kim et al. have shown effective delivery of the poorly water-soluble drug salicylazosulfapyridine (SSZ). These nanoparticles offer a controlled release effect, improve drug stability, and can be used for oral- and intestinal-targeted drug delivery systems. However their resilience in vivo should be tested later [136]. Gum karaya (GK)-functionalized AuNPs possess remarkable biocompatibility and stability. Incorporating these nanoparticles allows for targeted delivery of the anticancer drug gemcitabine hydrochloride (GEM) specifically to the tumor site. Notably, these nanoparticles have displayed substantial anticancer activity against human lung cancer cells [137]. AuNPs are easily prepared; however, their structural limitations necessitate an improvement in the polysaccharide-AuNPs drug delivery system’s structure to enhance the delivery process. Polysaccharide-AuNPs, characterized by a hollow structure, exhibit superior drug delivery capabilities and are employed for the loading and transporting of diverse anticancer drugs. Furthermore, the combination of AuNPs and cyclodextrin-grafted hyaluronic acid constructs results in the formation of innovative polysaccharide-AuNPs supramolecular conjugates (HACD-AuNPs) capable of delivering a wide range of antitumor drugs [138].

## 6. Drug Release from Polysaccharide-AuNPs

In recent years, there has been a significant increase in the attention given by researchers to responsive nano-drug release systems. This is primarily due to these systems’ unique triggering response mechanism. The primary purpose of these systems is to deliver drugs with precision to the tumor site, ensuring that treatment can be given on demand. By doing so, the occurrence of serious side effects resulting from early drug leakage and off-target drug delivery can be minimized. One of the driving factors behind the development of these systems is the recognition that the microenvironment at the site of infection differs from that of normal tissue. Specifically, there are differences in terms of substance concentration and composition. As a result, researchers have focused their efforts on creating responsive nano-drug release systems that both internal and external stimuli can trigger. Internal stimuli include pH and enzymes, while external stimuli include photothermal, laser-to-light, and ultrasound signals. Furthermore, researchers have also explored intelligent targeting methods that enable tumor-specific identification and targeted drug release. These advancements have significantly contributed to the responsive nano-drug release systems field. As a result, there is great promise for improved drug delivery in the future (Figure 7) [139].

Under the irradiation of light at specific wavelengths, the surface of gold nanoparticles generates the phenomenon of LSPR (Figure 7A), which gives the gold nanoparticles high photothermal conversion efficiency for use as photothermal and photosensitizer carriers [140]. Phototherapies, evolving rapidly as cancer treatment modalities, utilize light of different wavelengths to induce photochemical or photothermal changes within specific tissues [141]. Two commonly used forms of phototherapy are photodynamic therapy (PDT) and PTT. Both treatments involve the use of light and either exogenous or endogenous absorbers. These therapies aim to produce cytotoxic ROS or raise the local temperature in a targeted manner [142]. PDT and PTT techniques offer a significantly higher level of spatiotemporal control when compared to systemic therapies. This leads to a reduction in off-target toxicities.

Simultaneous PTT and PDT cancer therapy is accomplished by leveraging the gold nanoparticle’s thermal properties and its role as a drug delivery carrier for transporting the photosensitizer (PS) [140]. Zeng, J. et al. prepared a novel nanoplatform system consisting of porphyrin derivatives and Au NPs, in which modified chitosan was encapsulated on the surface of Au NPs through ligand exchange between sulfhydryl groups and Au. The chitosan-coated Au NPs (QCS-SH/Au NPs) were further coupled with endo-tetrakis(4-sulfophenyl)porphyrin (TPPS) to obtain porphyrin-conjugated Au-hybridized nanoparticles (TPPS/QCS-SH/Au NPs) Figure 7B briefly describes the preparation of TPPS/QCS-SH/Au NPs and their anticancer properties via PDT/PTT [143]. Notably, it has been observed that AuNPs functionalized with polysaccharides exhibit markedly improved effects in both PTT and PDT when compared to bare gold nanoparticles. This significant finding highlights the compelling potential for the application of polysaccharide-functionalized AuNPs in tumor therapy [143]. 

In recent years, PTT based on NIR light irradiation (in the wavelength range of 650–1400 nm) has gained widespread use due to its unique advantages. One of the main advantages is the excellent tissue penetration and minimal damage to healthy tissue it demonstrates. Specifically, NIR light is effective in penetrating the head of newborns to a depth of several millimeters [144,145]. Among various photothermal materials, gold nanostructures have gained significant attention in the field of PTT due to their remarkable LSPR properties. These properties are primarily responsible for inducing a photothermal effect.

Additionally, gold nanostructures possess excellent biocompatibility and exhibit strong light absorption in the NIR region. Consequently, they are considered highly suitable for PTT applications [146]. The induction of PTT for cancer treatment using gold nanoparticles was first published in 2003 [147]. PTT therapies, such as antibacterial and antitumor treatments, are widely used in various biological fields. Polysaccharide-AuNPs, in particular, have been found to induce a favorable immune response effectively at the tumor site. This leads to the conversion of an immunosuppressive TME into an immunostimulatory one. In PTT, NIR absorbers are typically employed to eradicate cancer cells. These absorbers efficiently convert light energy into heat, thereby increasing the local temperature of the tumor.

Consequently, the elevated temperature “burns” the cancer cells and destroys them. Using AuNPs conjugated to cisplatin, the antitumor effect can be enhanced by delivering and accumulating cisplatin at the tumor site. Under near-infrared light irradiation, this strategy maximizes the potential to treat tumors [148]. Liu et al. conducted a study in which poly (2-methylacrylic acid (3-amido-2,4,6-triiodobenzoic acid))/polyethyleneimine/hyaluronic acid modified AuNSs, referred to as PMATIB/PEI/Au/HA. These nanoshells were injected intravenously into mice with human mammary carcinoma MCF-7 cell-loaded tumors. The researchers observed that this injection significantly enhanced the contrast of CT imaging of the tumors. Moreover, this effect was achieved while selectively ablating the tumors through an increase in local temperature under NIR laser irradiation [149]. Hwa Seung Han et al. developed the hybridized hyaluronic acid gold nanocluster (AuNCs-HyNA). This material exploits the photothermal enhancement of photodynamic tumor ablation, achieved by incorporating the antitumor drug vitexoporfin. After reaching the tumor site via targeting hyaluronic acid, the material facilitates the decomposition of the hyaluronic acid, allowing the gold nanoclusters and the drug to enter the tumor tissue and exert a therapeutic effect [150]. The insertion of gold nanoclusters into carrageenan gum formed an antimicrobial hydrogel platform with a photothermal product, which showed sound antimicrobial effects against both Gram-positive and Gram-negative bacteria in vitro, and it is worthwhile to investigate other gold nanomaterials and whether the same antimicrobial effect can be produced against different strains of bacteria [151].

pH-responsive gold nanodrug carriers are widely used owing to the unique tumor microenvironment of tumor tissues, characterized by slightly acidic extracellular pH (~6.0–7.0) and intranuclear pH (~4.5–6.5) [152,153], it has proven to be an ideal tool for delivering therapeutic agents to lesions. In a study by Song et al., dextran and pullulan polysaccharides were used to encapsulate AuNRs, materials capable of self-destruction in the acidic environment of tumor cells and under conditions of high glutathione concentration. This approach enables targeted drug release specifically within tumor cells—the pH of the tissue changes after bacterial infection [154]. Zhang et al. utilized this property to design a pH-dependent nanocarrier for delivering curcumin to the site of bacterial infection. In their study, they modified the carrier with gold nanoparticles using chitosan. CH was added to improve the dispersibility of curcumin in an aqueous solution, while AuNPs were used to enhance the electrostatic binding ability of the carrier to the bacterial cell membrane. They developed a polysaccharide-AuNPs antibacterial platform with electrostatic-targeting photodynamic and photothermal antibacterial properties [155]. Shaimaa et al. [156] developed a pH-responsive nanocomposite hydrogel by synthesizing chitosan-grafted acrylamide monomers and incorporating gold nanoparticles. This nanocomposite hydrogel demonstrated a high sensitivity to changes in pH and exhibited rapid and efficient drug release, specifically at pH 7.4.

Furthermore, it exhibited cytotoxic solid effects against hepatocellular carcinoma cells [156]. High concentrations of glutathione (GSH) can cause a shift in the cellular environment, altering the pH from its physiological condition of 7.4 to pH 5.6. To address this issue, HA-modified dendrimers encapsulating AuNPs have been developed as active targeted drug delivery nanocarriers. These nanocarriers can efficiently load DOX by thiolation of the drug onto their surface, providing an effective treatment option for ovarian cancer. Furthermore, the loaded DOX can be explicitly released in the acidic tumor microenvironment with high GSH concentrations (pH 5.6) [157].

CAO was a biocompatible reductant to facilitate the green synthesis of CAO-AuNPs. Subsequently, the resultant CAO-AuNPs were employed as a delivery system for pH-triggered release of epirubicin (EPI). The EPI-CAO-AuNPs demonstrated a significant release of EPI under acidic conditions, which mimic the tumor microenvironment, while the release was negligible under physiological pH conditions in vitro. This study presents a novel approach to achieving pH-triggered freedom of anticancer drugs using the EPI-CAO-AuNPs system, marking the first instance of such findings. The developed EPI-CAO-AuNPs nanosystem exhibits excellent potential for pH-responsive delivery of antitumor agents [158]. The potential for toxicity remains the main obstacle to the current in vivo use of stimuli-responsive gold nanocomplexes.

In innovative targeted drug release systems, polysaccharides in gold nanocarriers can specifically bind to receptors overexpressed on tumor cell membranes. Zhang et al. prepared a galactose and alginose ligand-modified gold nanorod, a nanocomposite that effectively inhibited and killed Pseudomonas aeruginosa infections through lectin-targeting functionality and the near-infrared photothermal effect of AuNPs, a strategy that could be used to further generate antimicrobial agents, and its therapeutic effect on other infections should be verified [159]. Trastuzumab (Tmab) exhibits specificity in binding to the HER2 receptor. When combined with DOX and the monoclonal antibody trastuzumab, AuNPs can be used as a targeted drug delivery system for treating HER2+ cancers. This approach enables the delivery of the drug directly to the tumor site [160]. Folic acid (FA) binds explicitly to folate-targeting receptors, which are highly expressed on the surface of tumor cells. Therefore, by binding polysaccharide-AuNPs to FA, drug release at the tumor site can be enhanced. To achieve this, Panchanathan et al. prepared FA-coupled and DOX-loaded chitosan oligosaccharide-encapsulated AuNRs (FA-COS-TGA-GNRs-DOX). They found that under NIR light irradiation, these nanoparticles could accurately locate and destroy tumor tissues [161]. 2-[(Aminocarbonyl)amino]-5-(4-fluorophenyl)-3-thiophene carboxamide (TPCA-1) inhibits the NF-κB signaling pathway, thereby suppressing the transcription of TNF-α, IL-6, and other pro-inflammatory cytokines. AuNCs can be loaded with TPCA-1, and the binding of TPCA-1 to the HA-modified peptide allows HA to function as a targeting factor. This targeted delivery system facilitates the release of TPCA-1 and enzyme-responsive releasers. In the presence of an inflammatory microenvironment or upon encountering lysosomes, the HA undergoes degradation, leading to the release of TPCA-1. Thus, TPCA-1 exerts an anti-inflammatory effect and offers potential therapeutic benefits for the future treatment of rheumatoid arthritis (RA), but its anti-inflammatory activity and in vivo anti-RA mechanisms still require in-depth studies [162].

Besides the common pH-responsive drug release mechanism, new nano-drug delivery systems have been developed to achieve cascade-stimulated drug release. One of the commonly used cascade response stimulations is the pH/NIR co-stimulation. Xu et al. proposed a novel approach utilizing aldehyde/catechol functionalized hyaluronic acid (DAHA) and (HECS)-modified AuNRs platforms for combined chemo-photothermal therapy in breast cancer. Adjusting the polysaccharide-AuNPs onto the nanoplatforms could co-deliver AuNRs and DOX to the cancer cells. Moreover, introducing AuNRs enabled the nanoplatforms to possess pH/NIR dual-responsive drug release properties. This means the drug release can be triggered by either the pH level or the near-infrared light [163]. Cui et al. developed a novel gold coupling (AuNPs-NHN═Dox-mPEG) by modifying the positions of PEG and the drug on the gold nanosurface. This modification involved shielding DOX through polyethylene glycolization, which enhanced drug solubility, stability, and dispersion. The modified system also enabled responsive drug release in two steps upon exposure to an acidic environment in the lysosome. The sustained delivery of the modified system into the nucleus was intended to improve antitumor efficacy in vivo [164]. Xu et al. developed nanoplatforms highly responsive to pH, GSH, and hyaluronidase, targeting HER2 overexpression. These nanoplatforms possess dual-targeted receptors for HER2 and CD44, exhibiting desirable size and excellent dispersion. Moreover, when exposed to near-infrared radiation, MCF-7 cells efficiently generate reactive oxygen species and heat. The innovative combination therapy of PDT/PTT demonstrated a remarkable therapeutic effect on HER2-positive breast cancer. However, there is still a great challenge in reducing the non-specific nanoparticle accumulation in reticuloendothelial system organs, especially in the liver [165].

## 7. Future Perspectives and Conclusions

Polysaccharides are widely occurring substances and have diverse applications in adjuvant and antitumor therapies. Natural polysaccharides such as hyaluronic acid, chitosan, glycyrrhiza glabra, astragali polysaccharides, and ginseng polysaccharides are increasingly utilized in drug delivery for oncology treatments. Polysaccharide-AuNPs provide a versatile platform for delivering various drugs, including proteins, nucleic acids, and macromolecules. Modifying the surface of polysaccharide-AuNPs with different materials can improve drug targeting capability. The unique properties of AuNPs, including surface plasmon resonance, enable polysaccharide-AuNPs to exhibit a photothermal effect. This effect facilitates drug release in specific tissues upon NIR light irradiation.

Additionally, polysaccharide-AuNPs can respond to stimuli such as pH, enzymes, and lasers, enabling efficient drug release at specific locations and enhancing therapeutic efficacy. Although the drug delivery efficiency of polysaccharide-AuNPs is already considerable, surface modification can further strengthen this efficiency. Moreover, surface modification can facilitate targeting, enabling drug release in specific tissues. The response of polysaccharide-AuNPs to various stimuli also improves the efficiency of drug targeting and release. Ongoing research on natural polysaccharides supports the increasing use of natural polysaccharide-AuNPs in drug delivery and antitumor therapies. These advancements can significantly improve tumor treatment and enhance the therapeutic effects of drugs.

The utilization of polysaccharide-coated AuNPs (AuNPs) as drug delivery vehicles presents both opportunities and challenges. Polysaccharides such as chitosan and dextran are attractive materials for biomedical applications due to their favorable biocompatibility, biodegradability and low toxicity. When these polysaccharides are used to coat AuNPs, they can provide a stable and biocompatible platform for delivering therapeutic drugs. However, current applications of polysaccharide-coated AuNPs face challenges in terms of stability, size and shape control, targeted and selective drug delivery, immunogenicity and biocompatibility, and clinical trial safety. Although polysaccharide-coated AuNPs can provide stability and sustained drug release, there is no guarantee that nanoparticle stability will be maintained during in vivo storage and circulation. Therefore, controlling the size and shape of polysaccharide-coated AuNPs is critical to ensure proper drug loading and release profiles. In addition, functionalization of polysaccharide coatings may be required to achieve targeted drug delivery while avoiding off-target effects. Finally, there are fewer studies on the natural sources of plant polysaccharide-AuNPs, and it is worthwhile to further explore the potential herbal sources of polysaccharides and their applications in various aspects such as antitumor. Addressing the challenges of polysaccharide-coated AuNPs as drug delivery vehicles requires a multidisciplinary effort from materials science, pharmacology, and biomedical engineering to fully realize their potential for therapeutic applications.

## Figures and Tables

**Table 1:** Advantages and disadvantages of different nanocarriers.

Types of Nanomaterials	Advantages	Drawbacks
Dendritic polymers	good absorption and bioavailability, easy surface modifications [11], low toxicity, non-immunogenic and good pharmacokinetics [12]	complex arrangements, special reaction difficulties, and cationic dendritic polymers often exhibit high toxicity [11]
PLGA nanocarriers	biodegradable, biocompatible, slow-releasing [13]	poor binding of negatively charged drugs, rapidly cleared in vivo, unable to recognize cells, not selective [14,15]
PEI	excellent biocompatibility and high transfection efficiency [13]	potential cytotoxicity following intracellular accumulation [16]
Magnetic nanoparticles	superparamagnetic, reduces the distribution of cytotoxic substances in cells, and increases cellular uptake at targeted sites [17]	poor biocompatibility, hydrophobic surface, large specific surface area prone to cluster formation and thus embolization [17]
AuNPs	easy surface modification, size and shape control, photothermal effect, enhanced cellular uptake, increased drug toxicity, tumor growth inhibition, and selective drug targeting [18]	surface-dependent toxicity, poor stability under physiological conditions [19,20]
Mesoporous silica nanoparticles	highly biocompatible, controllable shape and size, allows for effective drug accumulation, improved drug stability and solubility [21]	not easy to store; restrictive drug loading; drug interactions in the mesoporous lumen [21]
Liposome	targeting tumors, promoting the mobility and durability of anticancer drugs, and improving pharmacokinetic and pharmacodynamic profiles of chemotherapeutic agents [9]	poor stability, low solubility, multidrug resistance, payload leakage, and phospholipid oxidation [22,23]
Carbon nanotubes	enhanced electrical conductivity and strength, easy to functionalize, easy to be absorbed by a variety of cells, unique optical properties can improve phototherapy [24]	non-biodegradable, poorly dispersed, and prone to aggregation and cytotoxicity [24]

**Table 2:** Methods for synthesizing AuNPs.

top-down	physical synthesis	physical fragmentation
		evaporation condensation
		plasma precipitation
		sputtering
bottom-up	chemical synthesis	Turkevich–Frens
		Brust–Schiffrin
	biosynthesis	

## Data Availability

Data are contained within the article.

## Abbreviations

APSs	astragalus polysaccharide
ARE	antioxidant response element
ASP	angelica sinensis polysaccharide
AuNCs	gold nanoclusters
AuNFs	gold nanoflowers
AuNPs	gold nanoparticles
AuNRs	gold nanorods
AuNSs	gold nanoshells
BBS	Brust-Schiffrin
BMP-2	bone morphogenetic protein 2
CAO	carrageenan oligosaccharide
CAT	catalase
CDT	chemodynamic therapy
CH	chitosan
CPPs	cell-penetrating peptides
CPT	cephalexin
CPT-11	irinotecan hydrochloride
Cs-AuNPs	chitosan capped gold nanoparticles
CT	computed tomography
CYP	chinese yam polysaccharides
DAHA	aldehyde/catechol-functionalized hyaluronic acid
DDS	drug delivery systems
DNA	deoxyribonucleic acid
DOX	doxorubicin
DPPH	1,1-Diphenyl-2-picrylhydrazyl radical 2,2-Diphenyl-1-(2,4,6-trinitrophenyl)hydrazyl
EGFR	epidermal growth factor receptor
EPI	epirubicin
EPR	enhanced permeability and retention effect
EPS	extracellular polysaccharides
FA	folic acid
GC	glycol chitosan
GEM	gemcitabine
GK	gum karaya
GOx	glucose oxidase
GSH	glutathione
GSH-Px	glutathione peroxidase
HA	hyaluronic acid
HACD	cyclodextrin-grafted hyaluronic acid
HBV	hepatitis B virus
HECS	hydroxyethyl chitosan
HER2	human epidermal growth factor receptor 2
HIV	human immunodeficiency virus
HPV	human papilloma virus
HyNA	hyaluronan nanoassembly
IL-6	interleukin-6
iNOS	inducible nitric oxide synthase
ITME	immunosuppressive tumor microenvironmen
LNT	lentinan
LSPR	local surface plasmonic resonance
MCF-7	Michigan Cancer Foundation-7
MDR	multidrug-resistant
MMP2	matrix metalloproteinase-2
M-M2pep	M2pep fusion peptide
M2-TAMs	M2-type tumor-associated macrophages
MTB	mycobacterium tuberculosis
MUA	11-mercaptoundecanoic acid
Nano-DDS	nanodrug delivery systems
MAPK	mitogen-activated protein kinase
NDs	nanodiamonds
NF-κB	nuclear factor kappa-B
NO	nitric oxide
NOS	nitric oxide synthase
Nrf2	nuclear factor erythroid 2-related factor 2
NIR	near-infrared
NK	natural killer
NPs	nanoparticles
OA	osteoarthritis
PA	pholiota adiposa
PAP-1a	pholiota adiposa polysaccharide
PCR	polymerase chain reaction
PDT	photodynamic therapy
PEI	polyetherimide
PEG	polyethylene glycol
PMATIB	poly(2-methylacrylic acid (3-amido-2,4,6-triiodobenzoic acid))
PS	photosensitizer
PSP	polysaccharide peptide
PtNPs	photothermal therapy
PTR	pleurotus tuber-regium
PTT	photothermal therapy
PTX	paclitaxel
QCS-SH/AuNPs	chitosan-stabilized gold nanoparticles
RhB	rhodamine B
ROS	reactive oxygen species
SB-SH	thiolsulfobetaine-stabilized
S-CYP	sulfated modifications chinese yam polysaccharides
-SH	-thiol
siRNA	small interfering RNA
SOD	superoxide dismutase
SPPs	the polysaccharide from Sargassum pallidum
SSZ	salicylazosulfapyridine
TC-AuNSs	thioglycolic chitosan-coated gold nanoshells
TCS	thiol chitosan
TLR4	toll-like receptor 4
Tmab	trastuzumab
TME	tumor microenvironment
TNF-α	tumor necrosis factor-α
TPCA-1	2-[(Aminocarbonyl)amino]-5-(4-fluorophenyl)-3-thiophenecarboxamide
TPPS	meso-tetrakis(4-sulphonatophenyl)porphyrin
TPT	topotecan
